# Evaluating the Safety and Efficacy of Naloxegol in Critically Ill Opioid-Induced Constipation Patients

**DOI:** 10.7759/cureus.41422

**Published:** 2023-07-05

**Authors:** Zeeshan M Rizwan, Rodney Garcia, Kristin Mara, Scott Nei

**Affiliations:** 1 Department of Pharmacy, Mayo Clinic, Rochester, USA; 2 Department of Nursing, Mayo Clinic, Rochester, USA; 3 Department of Biostatistics, Mayo Clinic, Rochester, USA

**Keywords:** critical care, efficacy study, icu patients, naloxegol, opioid induced constipation, pain management, safety study

## Abstract

Background

Opioid-induced constipation (OIC) has become more common in the intensive care unit (ICU) due to increased opioid utilization. Traditional laxatives often prove ineffective against OIC, leading to the increased utilization of naloxegol. However, further research is needed to confirm naloxegol's effectiveness and safety in critical care. This study aimed to explore the safety and efficacy of this intervention in critically ill OIC patients.

Methods

A single-center retrospective study was conducted on 353 patients who received one or more doses of naloxegol from January 1, 2019, to June 30, 2020, for OIC at a tertiary care center. The primary endpoint of this study was to evaluate serious adverse events such as reduced analgesic effect, gastrointestinal perforation, seizure, acute myocardial infarction (AMI), or ventricular arrhythmias using Naranjo Scale in critically ill patients. The secondary goal was to assess the efficacy of naloxegol, measured by the time of the first bowel movement.

Results

The average duration of naloxegol use was three days, with the first bowel movement occurring at an average of 11.3 hours. Furthermore, 59.8% of individuals had their first bowel movement within 20 hours of receiving naloxegol. There was a low level of causality between naloxegol use and adverse events such as gastrointestinal perforation, seizures, AMI, cardiovascular mortality, stroke, and ventricular arrhythmia. Additionally, reduction in analgesia showed no strong relationship with naloxegol use indicated by the Naranjo Scale assessment.

Conclusion

Naloxegol showed promising safety and efficacy profiles in treating OIC amongst critically ill patients, though our findings require further validation through prospective studies. This research paves the way for further investigation into naloxegol’s role in OIC management, emphasizing the necessity of personalized treatment strategies in critical care settings.

## Introduction

Constipation, a common complication in critically ill patients, is estimated to affect up to 70% of patients in the ICU setting [1]. Several factors, such as shock, dietary alterations, electrolyte disturbances, and common sedation and analgesia medications contribute to this issue [2]. Furthermore, as the Society of Critical Care Medicine recommends an analgesic-first approach for pain management, the use of opioids, which often exacerbates constipation, is frequent in critical care patients [3]. Understanding how opioids cause constipation, including activation of enteric μ-opioid receptors and inhibition of bowel function, is vital for treating opioid-induced constipation (OIC) [4,5]. Managing constipation is paramount to reestablishing normal gastrointestinal function, promoting enteral feeding, preventing bacterial translocation, relieving discomfort, and potentially shortening ICU stay [6,7].

Traditional approaches to managing constipation in critically ill patients utilize laxatives, including stimulants and stool softeners [8]. Nevertheless, these agents carry their own risks of local and systemic adverse effects and are often ineffective against OIC [9-11]. More recently, medications such as naloxegol, a peripherally acting µ-opioid receptor antagonist (PAMORA), have been introduced to address OIC, offering a new treatment option [12]. As a Food and Drug Administration (FDA)-approved treatment for OIC resistant to laxative therapy, naloxegol could provide critical relief to ICU patients [12-14].

Naloxegol offers a novel approach for controlling OIC by selective reversal of peripheral opioid effects without interfering with central pain management [14]. It should be noted that theoretically, naloxegol may precipitate opioid withdrawal by reduction of analgesia, potentially inducing arrhythmias, Major Adverse Cardiovascular Events (MACE), seizure, and gastrointestinal perforation [15-18]. Despite the aforementioned concerns, the application of naloxegol within critical care settings, where patient complications may be multifaceted, remains unverified due to limited evidence. As a result, further research is necessitated to establish a comprehensive understanding of naloxegol use in critically ill patients. We therefore retrospectively evaluated naloxegol's safety as our primary goal, using the Naranjo scale. Simultaneously, we also aimed to assess its efficacy, our secondary goal, in terms of time to the first bowel movement in 353 critically ill patients at Saint Mary's Hospital, Mayo Clinic from January 1, 2019, to June 30, 2020.

## Materials and methods

 We conducted a single-center, retrospective chart review of all adult patients who received naloxegol at the aforementioned institution from January 1, 2019, to June 30, 2020. The study was approved by the Mayo Clinic Institutional Review Board (IRB) prior to the initiation of the chart review process. Since the study was purely retrospective and did not involve experiments on humans or tissue sampling, informed consent was waived by the IRB. All methods were conducted following relevant guidelines and regulations.

All naloxegol doses were dispensed orally in tablet form, with the standard adult daily dosage as either 12.5 mg or 25 mg based on the creatinine clearance [12-14]. The demographic data gathered included the patient's age, gender, weight, category of ICU admission, and type of ICU admissions (Table 1), which were retrieved from the patient’s chart reviews and specific disease condition search criteria. Historical data included previous abdominal surgery, constipation, diverticulitis, coronary artery disease, diabetes, heart failure, and prior seizures. Patients presenting with evidence of pregnancy, active colitis from causes other than naloxegol, and infiltrative gastrointestinal (GI) tract malignancies were excluded from the study.

**Table 1:** Demographic and historical data for the critically ill patients ICU: intensive care unit, PMH: past medical history, CAD: coronary artery disease, GI: gastrointestinal

	Respiratory (N=31)	Neurological (N=1)	Cardiovascular (N=285)	GI (N=9)	Urology (N=1)	Trauma (N=17)	Carcinoma (N=9)	Total (N=353)	p-value
Age, Median (IQR)	50 (48, 65)	40	57 (45, 67)	59 (50, 66)	78	63 (60, 69)	50 (41, 65)	57 (47, 67)	0.27
Gender									0.42
Female	14 (45.2%)	0 (0.0%)	85 (29.8%)	2 (22.2%)	0 (0.0%)	5 (29.4%)	1 (11.1%)	107 (30.3%)	
Male	17 (54.8%)	1 (100.0%)	200 (70.2%)	7 (77.8%)	1 (100.0%)	12 (70.6%)	8 (88.9%)	246 (69.7%)	
Weight (kg), Median (IQR)	79 (70, 103)	55	87 (75, 106)	94 (82, 111)	80	82 (73, 87)	92 (65, 99)	86 (74, 105)	0.29
Type of ICU admitted to									<0.001
Missing	2	0	0	2	0	0	1	5	
Medical	24 (82.8%)	0 (0.0%)	5 (1.8%)	3 (42.9%)	0 (0.0%)	2 (11.8%)	0 (0.0%)	34 (9.8%)	
Surgical	5 (17.2%)	1 (100.0%)	280 (98.2%)	4 (57.1%)	1 (100.0%)	15 (88.2%)	8 (100.0%)	314 (90.2%)	
PMH - Constipation	20 (64.5%)	1 (100.0%)	70 (24.6%)	6 (66.7%)	0 (0.0%)	13 (76.5%)	4 (44.4%)	114 (32.3%)	<0.001
PMH - Abdominal surgery	0 (0.0%)	0 (0.0%)	0 (0.0%)	1 (11.1%)	0 (0.0%)	0 (0.0%)	0 (0.0%)	1 (0.3%)	<0.001
PMH - Diverticulitis	0 (0.0%)	0 (0.0%)	10 (3.5%)	0 (0.0%)	0 (0.0%)	2 (11.8%)	0 (0.0%)	12 (3.4%)	0.49
PMH - CAD	9 (29.0%)	0 (0.0%)	139 (48.8%)	4 (44.4%)	0 (0.0%)	9 (52.9%)	4 (44.4%)	165 (46.7%)	0.38
PMH - Diabetes	22 (71.0%)	0 (0.0%)	82 (28.8%)	1 (11.1%)	0 (0.0%)	7 (41.2%)	0 (0.0%)	112 (31.7%)	<0.001
PMH - Seizures	4 (12.9%)	0 (0.0%)	22 (7.7%)	1 (11.1%)	0 (0.0%)	0 (0.0%)	1 (11.1%)	28 (7.9%)	0.81
PMH - Heart Failure	8 (25.8%)	0 (0.0%)	218 (76.5%)	3 (33.3%)	0 (0.0%)	6 (35.3%)	4 (44.4%)	239 (67.7%)	<0.001

To characterize the data acquired, we employed descriptive statistics. Furthermore, in this descriptive analysis, the Naranjo Adverse Drug Reaction Probability Scale also known as Naranjo Scale was used to describe the major adverse events (Table 2). This scale is designed to standardize the assessment of causality for adverse drug reactions. It consists of 10 questions with the following answers to choose from: "yes," "no," and "do not know." The scores for each of these questions range between −1 and +2. The total sum of the scores ranges from -4 to +13 and helps determine the probability of the cause of the adverse reaction. The adverse drug reaction is considered: definite if the score is 9 or higher, probable if 5 to 8, possible if 1 to 4, and doubtful if 0 or less [19].

**Table 2:** Naranjo Scale - adverse drug reaction probability scale

Question	Yes	No	Do Not Know	Score
1. Are there previous conclusive reports on this reaction?	+1	0	0	
2. Did the adverse event appear after the suspected drug was administered?	+2	-1	0	
3. Did the adverse event improve when the drug was discontinued or a specific antagonist was administered?	+1	0	0	
4. Did the adverse event reappear when the drug was readministered?	+2	-1	0	
5. Are there alternative causes that could on their own have caused the reaction?	-1	+2	0	
6. Did the reaction reappear when a placebo was given?	-1	+1	0	
7. Was the drug detected in blood or other fluids in concentrations known to be toxic?	+1	0	0	
8. Was the reaction more severe when the dose was increased or less severe when the dose was decreased?	+1	0	0	
9. Did the patient have a similar reaction to the same or similar drugs in any previous exposure?	+1	0	0	
10. Was the adverse event confirmed by any objective evidence?	+1	0	0	

Definitions

Critically ill patients were identified as those admitted to the Medical ICU, Surgical ICU, Anesthesia ICU, and Cardiovascular ICU.

OIC was defined as a change when initiating opioid therapy from baseline bowel habits that are characterized by any of the following: reduced bowel movement frequency, development or worsening of straining to pass bowel movements, a sense of incomplete rectal evacuation, or harder stool frequency [20].

The reduction in analgesia was defined by an increase in opioid requirement at 24 hours and 24 to 48 hours after the administration of naloxegol.

Laxative use included therapy with agents such as osmotic laxatives, stool softeners, or stimulants used for any duration of time.

Bowel obstruction is a blockage that keeps food or liquid from passing through the small or large intestine and gastric perforation is a full-thickness injury to the wall of the stomach [21].

## Results

As seen in Table 3 the mean total morphine equivalent dose (MED) of opioids used 24 hours before the administration of the naloxegol dose was 95 MED and the mean total MED of opioids used 24 hours and 48 hours post-dose was 64 MED and 38 MED, respectively. We note that the opioid requirement in the respiratory ICU patients increased from a mean of 180 MED (0, 585) 24 hours prior to naloxegol administration, to 229 MED 24 hours post naloxegol administration.

**Table 3:** Adverse drug reaction data for the critically ill patients MED: morphine equivalent dose, GI: gastrointestinal, AMI: acute myocardial infarction, MACE: major adverse cardiac events, BM: bowel movement

	Respiratory (N=31)	Neurological (N=1)	Cardiovascular (N=285)	GI (N=9)	Urology (N=1)	Trauma (N=17)	Sarcoma/Carcinoma (N=9)	Total (N=353)	p-value
Total Dose in MED of opioid used 24 hours prior to T, Median (IQR)	180 (0, 585)	16	90 (24, 226)	137 (56, 263)	15	63 (15, 206)	130 (50, 240)	95 (24, 240)	0.23
Total Dose in MED of opioid used 24 hours after T, Median (IQR)	229 (0, 656)	552	60 (8, 189)	100 (30, 138)	146	55 (23, 115)	113 (38, 340)	64 (8, 221)	0.26
Total Dose in MED of opioid used 24-48 hours after T, Median (IQR)	100 (0, 495)	368	31	23 (8, 95)	5	40 (15, 129)	208 (45, 295)	38 (0, 180)	0.24
Time from last documented BM to Naloxegol dose (No. hours prior to T), Median (IQR)	28 (4, 85)	42	51 (17, 83)	39 (13, 122)	2.3	19 (3, 45)	29 (22, 36)	45 (14, 83)	0.16
Time from administration to first BM (No. of hours after T), Median (IQR)	5 (2, 23)	4.8	13 (3.3, 36.3)	3 (2, 18.3)	35	3.8 (1.5, 10)	25 (2.5, 40.8)	11.3 (3, 35)	0.10
Duration of Naloxegol administration (Days from T to cessation), Median (IQR)	3 (1, 4)	3	3 (1, 5)	4 (1, 6)	6	1 (1, 3)	2 (1, 3)	3 (1, 4)	0.42
Reduction in analgesic effect	10 (32.3%)	1 (100.0%)	94 (33.0%)	1 (11.1%)	1 (100.0%)	5 (29.4%)	5 (55.6%)	117 (33.1%)	0.23
GI perforation	0	0	0	0	0	0	0	0	
Seizure	0	0	0	0	0	0	0	0	
MACE: AMI	0	0	2 (0.7%)	0	0	0	0	2 (0.6%)	0.99
MACE: Cardiovascular death	0	0	0	0	0	0	0	0	
MACE: Stroke	0	0	0	0	0	0	0	0	
Ventricular Arrhythmias	1 (3.2%)	0	4 (1.4%)	0	0	2 (11.8%)	0	7 (2.0%)	0.15

The majority of the patients in our study, accounting for 314 (88.9%), were admitted due to surgical reasons, and 34 (11.1%) patients were admitted for medical reasons (Table 4). Among these, 117 patients (33.1%) experienced a reduction in analgesic effect. Two patients (0.6%) in the cardiovascular group had an acute myocardial infarction (AMI) with a Naranjo score of zero, and seven patients (2.0%) experienced ventricular arrhythmia with one patient scoring one on the Naranjo Scale.

**Table 4:** Distribution of patients by reduction of analgesic effect a Per 10 units b Per 50 units ICU: intensive care unit, PMH: past medical history, CAD: coronary artery disease, MED: morphine equivalent dose, BM: bowel movement

	Reduction in analgesia			
	Yes (N=117)	No (N=236)	Odds Ratio (95% CI)	p-value
Age, Median (IQR)	56 (43, 64)	60 (48, 67)	0.85 (0.73-0.99)^a^	0.036
Gender				
Female	42 (35.9%)	65 (27.5%)	1.47 (0.92-2.37)	0.11
Male	75 (64.1%)	171 (72.5%)	Reference	
Weight (kg), Median (IQR)	82 (71, 109)	88 (76, 104)	0.96 (0.87-1.05)^a^	0.35
Type of ICU admitted to				0.93
Missing	2	3		
Medical	11 (9.6%)	23 (9.9%)	Reference	
Surgical	104 (90.4%)	210 (90.1%)	1.03 (0.46-2.27)	0.95
Category of ICU admission				
Respiratory	10 (8.5%)	21 (8.9%)	0.99 (0.45-2.18)	0.98
Neurological	1 (0.9%)	0 (0.0%)	6.41 (0.07-634.67)	0.43
Cardiovascular	94 (80.3%)	191 (80.9%)	Reference	
Gastrointestinal	1 (0.9%)	8 (3.4%)	0.36 (0.06-2.27)	0.28
Urology	1 (0.9%)	0 (0.0%)	6.41 (0.07-634.67)	0.43
Trauma	5 (4.3%)	12 (5.1%)	0.89 (0.31-2.58)	0.83
Sarcoma/Carcinoma	5 (4.3%)	4 (1.7%)	2.48 (0.65-9.42)	0.18
PMH - Constipation	39 (33.3%)	75 (31.8%)	1.08 (0.67-1.73)	0.76
PMH - Diverticulitis	6 (5.1%)	6 (2.5%)	2.09 (0.66-6.62)	0.21
PMH - CAD	50 (42.7%)	115 (48.7%)	0.80 (0.51-1.25)	0.34
PMH - Diabetes	38 (32.5%)	74 (31.4%)	1.05 (0.65-1.70)	0.84
PMH - Seizures	12 (10.3%)	16 (6.8%)	1.54 (0.71-3.36)	0.27
PMH - Heart Failure	77 (65.8%)	162 (68.6%)	0.90 (0.56-1.45)	0.66
Total Dose in MED of opioid used 24 hours prior to T, Median (IQR)	104 (35, 232)	92 (10, 257)	0.99 (0.95-1.03)^b^	0.48
Total Dose in MED of opioid used 24 hours after T, Median (IQR)	160 (68, 413)	30 (0, 122)	1.15 (1.08-1.23)^b^	<0.001
Total Dose in MED of opioid used 24-48 hours after T, Median (IQR)	180 (52, 375)	8 (0, 66)	1.25 (1.12-1.38)^b^	<0.001
Time from last documented BM to Naloxegol dose (No. hours prior to T), Median (IQR)	42 (16, 81)	50 (13, 83)	1.01 (0.96-1.07)^a^	0.58
Time from administration to first BM (No. of hours after T), Median (IQR)	17 (4, 41)	10 (3, 30)	1.17 (1.08-1.26)^a^	<0.001
Duration of Naloxegol administration (Days from T to cessation), Median (IQR)	3 (2, 5)	3 (1, 4)	1.03 (0.99-1.07)	0.14

**Figure 1:**
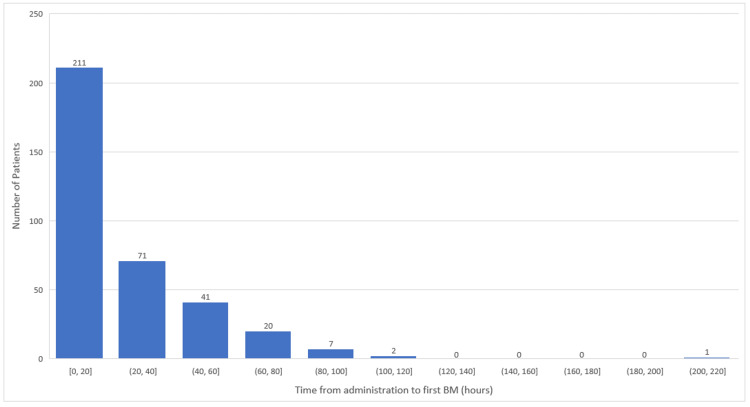
Time to first BM after the administration of naloxegol BM: bowel movement

The mean usage duration of naloxegol was observed to be three days, and the time from administration to the first bowel movement was 11.3 hours as seen in Table 3. It is also noted that 211 patients (59.8%) had their first bowel movement within the first 20 hours of taking naloxegol (Figure 1). In our analysis, we observed that the Naranjo score for the adverse effects ranged from 0 to 4 (Table 5). No adverse event achieved a score higher than four. It was also noted that the Naranjo score was consistently zero for categories such as GI perforation, seizure, AMI, cardiovascular death, and stroke and that only one patient reported ventricular arrhythmia with a Naranjo score of 1, indicating a very low possibility that these were adverse medication reactions. The frequency of the "reduction in analgesic effect" category varied across Naranjo score groups with the highest incidence of this event observed at the score of 0 (n=234), followed by a score of 1 (n=84). Fewer events were reported at scores 2 (n=6), 3 (n=8), and 4 (n=21).

**Table 5:** Distribution of Naranjo score among different adverse outcomes post-naloxegol administration AMI: acute myocardial infarction

Naranjo Score (Total)	Reduction in analgesic effect	Gastrointestinal perforation	Seizure	AMI	Cardiovascular death		Ventricular arrhythmias
0	234	353	353	353	353	353	352
1	84	0	0	0	0	0	1
2	6	0	0	0	0	0	0
3	8	0	0	0	0	0	0
4	21	0	0	0	0	0	0

## Discussion

In our study, the average duration of naloxegol usage was found to be three days, with the average time to the first bowel movement post-administration being 11.3 hours. The first bowel was experienced within the first 20 hours after initiating naloxegol in 211 patients (59.8%). This rapid and effective response may be attributed to the pharmacokinetic properties of naloxegol as stated in the package insert. Specifically, naloxegol is absorbed rapidly with peak concentrations (Cmax) achieved in less than 2 hours. A secondary plasma concentration peak typically occurs approximately 0.4 to 3 hours after the first peak, which aligns with the observation that a significant proportion of our patients had their first bowel movement within the first 20 hours post-administration. Furthermore, the elimination half-life of naloxegol is between 6 and 11 hours, which could provide further explanation for the average three-day usage duration observed in our patient cohort [15].

The KODIAC-04 and KODIAC-05 Phase 3 trials provide information on possible side effects associated with naloxegol [14]. These may include a decrease in analgesia, GI perforation, seizure, AMI, stroke, and ventricular arrhythmia. Within our patient cohort, we noted adverse events in 126 patients following the administration of naloxegol.

Based on the results and the Naranjo score distribution we can infer that there is a low level of causality between naloxegol use and the adverse events investigated in this study. We observed various frequencies of the Naranjo score for reduction in analgesia, but none indicated a strong relationship with naloxegol usage thereby showing that the association between naloxegol and reduced analgesia is unknown. In this study, reduction in analgesia was gauged by an increase in opioid requirement at 24 and 24 to 48 hours after naloxegol administration which can be influenced by several factors outside other than drug administration, such as the intensity and type of surgery, sedation status, individual patient physiology, and pain tolerance. The potential influence of these confounding factors may be reflected in the distribution of scores. We also observed that the total MED was higher in the medical ICU patients as compared to the surgical patients. This could be attributed to the fact that the majority of the medical ICU patients were admitted due to respiratory complications, necessitating the use of high-dose opioids during the course of mechanical ventilation. As for severe adverse events, we recorded two instances of AMI and seven ventricular arrhythmias within the cardiovascular patient group. These occurrences could plausibly be linked to each patient's medical history and the state of their disease, as opposed to the administration of naloxegol.

We also found significant variance in the time to first bowel movement post-naloxegol administration, 17 hours versus 10 hours between the groups that experienced a reduction in analgesic effect contrasted with the group that did not (OR 1.17 (1.08-1.26) p <0.001) as seen in Table 4. This observation can be supported by the significant increased MED at 24 hours (OR 1.15 (1.08-1.23) p < 0.001) and 24 to 48 hours (OR 1.25 (1.12-1.38) p< 0.001) post-naloxegol administration in the group that experienced analgesia reduction compared to the group that did not. It is worth noting that while these differences were observed, other potential confounding influences could also contribute to this effect, necessitating careful interpretation of these results.

While our study has yielded some beneficial findings, it is important to understand its limitations. The study being conducted in a single center might limit the generalizability of the findings due to the differences in patient demographics and healthcare practices. The inherent nature of retrospective studies may introduce potential biases and unmeasured confounding factors, potentially limiting the precision and accuracy of our analysis. Moreover, the presence of confounding factors presents another challenge. An analysis for confounders could isolate the effects of naloxegol and other variables. This would ensure a more accurate understanding of the drug's influence on ADRs, thereby yielding more reliable results. The use of the Naranjo score as a causality assessment tool has its own limitations as it might not accurately represent the true cause-effect relationship. The scoring system is subjective and relies on clinical judgment, which can introduce bias and variability among different evaluators.

## Conclusions

Our study offers considerable insight into the effectiveness and safety characteristics of naloxegol among critically ill patients. We found that naloxegol exhibits a generally safe profile for use in critically ill OIC patients, mainly concerning potential adverse events such as GI perforation, seizures, AMI, stroke, cardiovascular death, and ventricular arrhythmias. The acknowledgment of potential confounding factors that may impact reduction in analgesia reflects the complexity involved in managing pain in critically ill patients and highlights the need for personalized therapeutic interventions. Our research also emphasizes the essential role of pharmacovigilance within the critical care environment, serving as a substantial foundation for future investigations, particularly those seeking to utilize enhanced tools for more accurate causality assessment. The findings of our study also pave the way for new hypotheses and prospective studies with similar outcome metrics aiming to refine the understanding and management of OIC in critically ill patients. Further prospective studies are warranted to ascertain the true association of naloxegol and adverse drug reactions with in-depth analyses of the safety and efficacy of naloxegol.
